# Shift in Food Intake and Changes in Metabolic Regulation and Gene Expression during Simulated Night-Shift Work: A Rat Model

**DOI:** 10.3390/nu8110712

**Published:** 2016-11-08

**Authors:** Andrea Rørvik Marti, Peter Meerlo, Janne Grønli, Sjoerd Johan van Hasselt, Jelena Mrdalj, Ståle Pallesen, Torhild Thue Pedersen, Tone Elise Gjøtterud Henriksen, Silje Skrede

**Affiliations:** 1 Department of Biological and Medical Psychology, University of Bergen, Bergen 5009, Norway; janne.gronli@uib.no (J.G.); jelena.mrdalj@uib.no (J.M.); torhildp@hotmail.com (T.T.P.); 2 Groningen Institute for Evolutionary Life Sciences, University of Groningen, 9700 CC Groningen, The Netherlands; p.meerlo@rug.nl (P.M.); sjoerdvanhasselt@live.nl (S.J.v.H.); 3 College of Medical Sciences, Washington State University, Spokane, WA 99210, USA; 4 Sleep and Performance Research Center, Washington State University, Spokane, WA 99210, USA; 5 Norwegian Competence Center for Sleep Disorders, Haukeland University Hospital, Bergen 5021, Norway; 6 Department of Psychosocial Science, University of Bergen, Bergen 5015, Norway; staale.pallesen@uib.no; 7 Section of Psychiatry, Department of Clinical Medicine, Faculty of Medicine and Dentistry, University of Bergen, Bergen 5021, Norway; tone.elise.gjotterud@helse-fonna.no; 8 Division of Mental Health Care, Valen Hospital, Fonna Local Health Authority, Valen 5451, Norway; 9 Dr. Einar Martens Research Group for Biological Psychiatry, Center for Medical Genetics and Molecular Medicine, Haukeland Univeristy Hospital, 5021 Bergen, Norway; silje.skrede@uib.no

**Keywords:** shift work, night work, animal model, metabolism, circadian rhythmicity, gene expression, body temperature, body weight, food intake

## Abstract

Night-shift work is linked to a shift in food intake toward the normal sleeping period, and to metabolic disturbance. We applied a rat model of night-shift work to assess the immediate effects of such a shift in food intake on metabolism. Male Wistar rats were subjected to 8 h of forced activity during their rest (ZT2-10) or active (ZT14-22) phase. Food intake, body weight, and body temperature were monitored across four work days and eight recovery days. Food intake gradually shifted toward rest-work hours, stabilizing on work day three. A subgroup of animals was euthanized after the third work session for analysis of metabolic gene expression in the liver by real-time polymerase chain reaction (PCR). Results show that work in the rest phase shifted food intake to rest-work hours. Moreover, liver genes related to energy storage and insulin metabolism were upregulated, and genes related to energy breakdown were downregulated compared to non-working time-matched controls. Both working groups lost weight during the protocol and regained weight during recovery, but animals that worked in the rest phase did not fully recover, even after eight days of recovery. In conclusion, three to four days of work in the rest phase is sufficient to induce disruption of several metabolic parameters, which requires more than eight days for full recovery.

## 1. Introduction

Working shifts is common in modern societies [1], despite the fact that chronic night-shift work is linked to an increased risk for a wide variety of diseases, including metabolic disorders such as obesity and diabetes [2,3,4,5,6,7,8]. 

The regulation of daily rhythmicity in physiology and behaviour is closely intertwined with the regulation of metabolism. The circadian clock in the suprachiasmatic nuclei (SCN) of the hypothalamus influence other hypothalamic regions and peripheral tissues involved in regulation of feeding behaviour, metabolism, energy storage, and energy breakdown [9]. 

An important function of the endogenous circadian system is that it allows for anticipation of meals, and thereby facilitates efficiency in metabolic regulation [10]. The activity of organs involved in food processing and energy metabolism—including the digestive tract [11], liver [12,13], and pancreas [14]—shows clear circadian rhythmicity. Food intake is thought to be one of the primary zeitgebers (time-givers) for the coordination and timing of these rhythms [15]. 

Night-shift workers are exposed to conflicting zeitgebers, imposed by changes in food intake and activity patterns, as well as nocturnal light exposure [16,17,18]. Consequently, during night-shift work, endogenous circadian rhythms become desynchronized, as conflicting signals are sent to different tissues and organs. Often, the desynchronization is prolonged, as entrainment of rhythms in different tissues occurs at different rates [10]. 

The effects of working at night are immediate, and although humans may partially adapt to the night-shift, many physiological systems fail to adjust [19]. In Norway and Europe generally, night workers commonly work three to four night shifts in a row. Data from a simulated night-shift study in humans indicate that the metabolic effects are most pronounced during the initial two days, followed by compensatory mechanisms triggered by negative metabolic effects [20]. Ribeiro and colleagues induced a 9 h phase delay in a human simulated shift work study. Controlling energy and macronutrient intake, they showed that plasma levels of free fatty acids and triglycerides were reduced postprandially, indicative of poor metabolic regulation, but that this reduction was partially normalized on the third day following the phase delay [20].

Animal models of shift work may provide important clues to the mechanisms by which shift work causes metabolic disruption. Although several animal models indicate marked metabolic changes due to rest-work [21], only models involving relatively extreme rest-work schedules have been applied so far. The early metabolic effects of simulated night-shift work have yet to be investigated in animal models. 

In the present study, we utilized a rat model of shift work to investigate the early effects of simulated night work on metabolism. We hypothesized that a shift in feeding rhythm during work hours occurs during a four-day rest-work schedule, as a sign of metabolic compensation. Specifically, we proposed that compensatory mechanisms triggered by negative metabolic effects would be displayed in liver gene expression due to a shift in the timing of food intake. 

## 2. Materials and Methods 

### 2.1. Ethical Approval

This project was approved by the Norwegian Animal Research Authority (permit number: 2012463) and performed according to Norwegian laws and regulations, and The European Convention for the Protection of Vertebrate Animals used for Experimental and Other Scientific Purposes.

### 2.2. Animals and Housing

Adult male rats (*n* = 40, Wistar, NTac:WH, Taconic, Silkeborg, Denmark) weighing approximately 300 g at arrival, were group housed in individually ventilated cages (IVC, Tecniplast, Buggugitate, Italy, 75 air changes/h) type IV (480 × 375 × 210 mm, 1500 cm^2^). After surgery and during the experiment, animals were single housed (IVC cage type III, 425 × 266 × 185 mm, 800 cm^2^). The study was performed under a 12 h light/12 h dark (LD) cycle (lights on at 06:00, zeitgeber time 0; ZT0). Lights were gradually dimmed on and off over a period of 1 h, and were fully on at 07:00 and fully off at 19:00. Food (rat and mouse No. 1, Special Diets Services, Witham, Essex, UK) and water were provided ad libitum throughout the experiment.

### 2.3. Study Design

**Figure 1:**
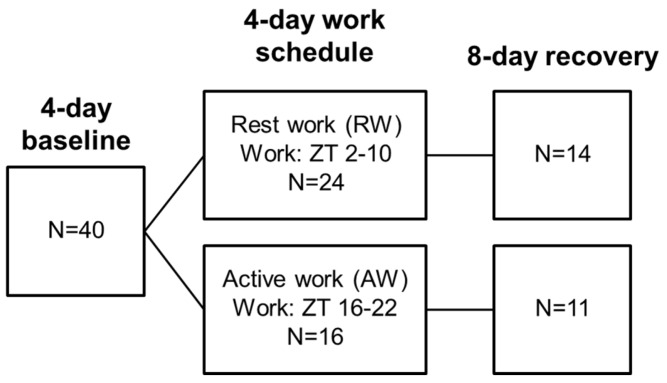
Overview of the study design. Animals were monitored for four baseline days, and a four-day work schedule during which animals were exposed to either rest-work or active work. The work schedule was followed by eight days of recovery. After at least five weeks of recovery, a subset of animals underwent a three-day work schedule (not shown) before euthanasia and tissue harvest from experimental animals and undisturbed time-matched controls.

To assess changes in food timing, rats were randomly assigned to one of two treatment groups, either rest-work (RW, *n* = 24) or active-work (AW, *n* = 16). The experiment first consisted of four days of undisturbed baseline, followed by a four-day work period. A subset of animals was monitored during eight days of undisturbed recovery (RW *n* = 14, AW *n* = 11). See Figure 1 for a graphical overview of the study design. A shift in feeding rhythm of RW animals was observed during work session three. For assessment of changes in metabolic gene expression in the liver during this shift, another experiment was conducted in a subset of animals (RW *n* = 10, AW *n* = 10) after five weeks of recovery from the initial four-day shift-work period. Since the animals were still young adults [22], we consider it unlikely that ageing affected the results of this second experiment. The animals were randomly assigned to three consecutive days of either AW or RW as described above, euthanized after the third work session, and compared to time-matched non-working controls (*n* = 5, each condition).

### 2.4. Simulated Shift Work Procedure

To mimic human shift work and compare this with normal daytime work, rats were exposed to forced activity for 8 h per day, centred either during the rats’ normal active phase (AW; ZT 14-22) or during the rats’ normal rest phase (RW; ZT 2-10), as described previously [23]. Forced activity was achieved by placing the rats in automatically rotating wheels (Rat Running Wheel, TSE running wheel system, Bad Homburg, Germany; 24 cm diameter; 3 rpm; 1440 revolutions or 1.086 km of linear distance per 8 h session). Food and water was available ad libitum. Rotating wheels, feeders, and water bottles were cleaned after each work session with a 5% ethanol solution. Between sessions, animals were housed in their home cages. 

### 2.5. Telemetric Recording and Analyses of Body Temperature 

Rats were implanted with transmitters (Physiotel, Data Sciences International, St. Paul, MN, USA) for continuous wireless recording of body temperature, as previously described [24]. In brief, animals were anaesthetized with subcutaneous injection of a mixture of fentanyl 0.277 mg/kg, fluanizone 8.8 mg/kg, and midazolam 2.5 mg/kg (Hypnorm, Janssen, Beerse, Belgium; Dormicum, Roche, Basel, Switzerland; Midazolam Actavis, Actavis, Parsippany-Troy Hills, NJ, USA), and the transmitters were placed in subcutaneous pockets in the dorsomedial lumbar region (4ET transmitters) or in the neck region (F40-EET transmitters). Animals were allowed to recover for 14 days before entering the experiment. Body temperature was recorded every 10 s, at 50 Hz sampling rate, and signals were collected with Dataquest ART software (version 4.1, Data Sciences International, St. Paul, MN, USA). Chronos-Fit software (Heidelberg University, Heidelberg, Germany) [25] was used for linear analyses of body temperature. From the linear analysis, 24 h mean, 12 h rest phase mean (lights on; ZT 12–24), and 12 h active phase mean (lights off; ZT 0–12) were calculated.

### 2.6. Body Weight and Food Intake Measurements

At baseline, all animals were weighed to assess 24 h and four-day body weight change. Baseline food and water intake were monitored across an 8 h window equal to the length of one work session, and across a 16 h window equal to the time between each work session. During the four-day work period, body weight change, food intake, and water intake were monitored for each 8 h work session and the 16 h between the work sessions. During the eight-day recovery phase, body weight change was monitored every four days.

### 2.7. Assessment of Metabolic Gene Expression in the Liver

Following the third work session, animals were fasted for 2 h to avoid the immediate effects of food intake on gene expression, anaesthetized with isoflurane, and sacrificed by decapitation. AW were sacrificed at ZT0, before the transition from dark to light phase, and RW at ZT12, before the transition from light to dark phase. A separate group of undisturbed animals never exposed to simulated work were used as time-matched controls, and sacrificed at the same zeitgeber times as experimental animals (AW control: ZT0; and RW control: ZT12). Liver tissue was harvested, flash frozen, and stored at −80 °C until analysis. Samples were homogenized using a TissueLyser (Qiagen, Valencia, CA, USA). RNA extraction was performed using a 6100 Nucleic acid PrepStation (Applied Biosystems, Foster City, CA, USA). A total of 20 ng RNA was transcribed to cDNA using the High Capacity RNA-to-cDNA kit (Applied Biosystems). Real-time polymerase chain reaction (PCR) was run on the Applied Biosystems 7900 Real-Time PCR System, with each sample run in triplicate. Relative gene expression levels were determined using the comparative ΔCt method, using β-actin (Actb) and ribosomal protein lateral stalk subunit P0 (Rplp0) as endogenous controls. Sequence names, main function, accession numbers, and primer sequences are shown in Table 1.

### 2.8. Statistical Analyses

Statistical analyses were conducted using STATA (release 14; StataCorp LP, College Station, TX, USA). Baseline food intake and body weight were compared between groups using student’s *t*-test (two-tailed). For all other statistical analyses of food intake, body weight, and body temperature, we used mixed model analysis using restricted maximum likelihood estimation with the unstructured covariance between random effects. Where significant effects were observed, pairwise comparisons of groups at each time point were performed as well as comparing each day to baseline. Difference in gene expression (fold change) between groups was evaluated using student’s *t*-test (two-tailed). Statistical significance was accepted at *p* < 0.05.

## 3. Results

### 3.1. Baseline Parameters 

At baseline, the two groups on average had similar body weight and food intake (*p* > 0.09 in all cases). The absolute baseline food intake across 24 h was 23.1 (±0.53 g) for RW and 21.36 (±0.70 g) for AW. Eight hours baseline food intake was 4.39 (±0.37 g) for RW and 8.54 (±0.70 g) for AW. Moreover, there were no significant differences in body temperature parameters (24 h mean, active phase mean, rest phase mean; *p* > 0.10 in all cases). Baseline day three was used as baseline reference in the subsequent analyses. 

### 3.2. Feeding and Body Temperature Rhythms, during and after One Rest-Work Period

**Figure 2:**
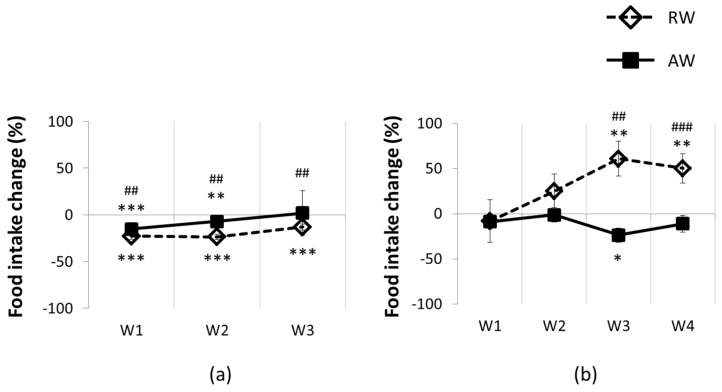
Food intake across one shift work period for active workers (AW) and rest-workers (RW): (**a**) 24 h food intake; (**b**) 8 h food intake (during work session). Data are shown as percentage change relative to baseline. Error bars indicate SEM. W1–4 indicates work days 1 to 4. W4 not included in 24 h food intake due to missing measurements. * *p* < 0.05; ** *p* < 0.01; *** *p* < 0.001, compared to baseline. ## *p* < 0.01; ### *p* < 0.001, between groups.

In RW, total 24 h food intake was significantly reduced on all four work days compared to baseline (*p* < 0.001 for all days; Figure 2a), but not compared to AW. RW food intake gradually increased across the first two 8 h work sessions. On day three, food intake stabilized, and was significantly increased in the 8 h work sessions on both days three and four (*p* < 0.02; Figure 2b). Hence, RW shifted the timing of food intake on work day three. This shift was also significant compared to AW food intake during the work session on days three and four (*p* < 0.01, both work sessions; Figure 1b). Between work sessions, food intake was decreased on all work days (*p* < 0.001, all days) compared to baseline. 

**Figure 3:**
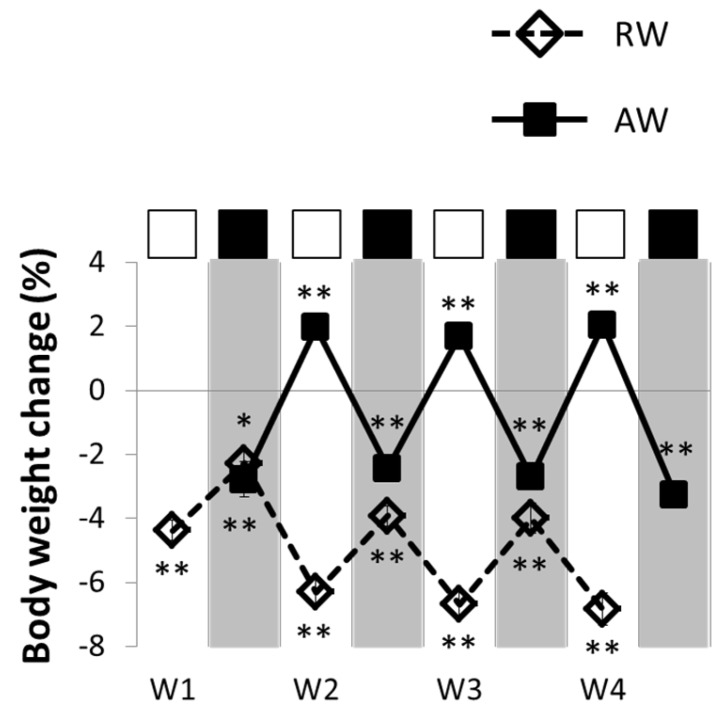
Body weight change during and between work sessions for active workers (AW) and rest-workers (RW). White rectangles indicate work hours for RW. Black rectangles indicate work hours for AW. Data are shown as percentage change relative to baseline. Shaded bars indicate lights off (active phase). Error bars indicate SEM. W1–4 indicates work days 1 to 4. * *p* < 0.05; *** *p* < 0.001, compared to baseline.

RW body weight dropped and remained below baseline across the whole four-day rest-work period (Figure 3). The reduced body weight was evident across 24 h, and during the 8 h work sessions. RW gained less weight between the work sessions, both compared to baseline (*p* < 0.001, all work days) and to AW (*p* < 0.008, all work days). The most pronounced body weight loss was observed during work day two, before body weight loss appeared to attenuate (see Figure 3). 

**Figure 4:**
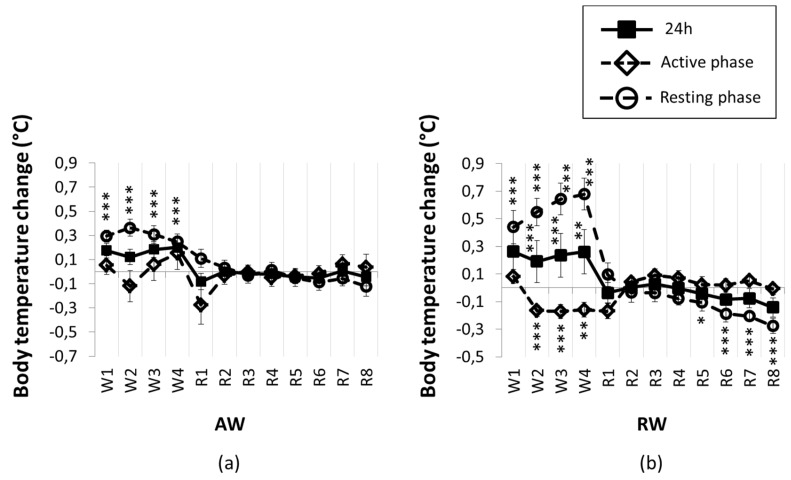
Mean body temperature during one shift work period (W1–4) and recovery (R1–8) for (**a**) active workers (AW) and (**b**) rest-workers (RW). Data are shown as mean percentage change relative to baseline. Error bars indicate SEM. W1–4 indicate work days 1 to 4. ** *p* < 0.01; *** *p* < 0.001, compared to baseline.

In RW, mean 24 h body temperature during the four-day rest-work period did not significantly differ from baseline or AW. However, RW body temperature was elevated during the 12 h rest phase (which now included the 8 h work session; *p* < 0.001 for all four work days), and was reduced during the 12 h active phase on work days two to four (*p* < 0.003 all three work days, Figure 4b). The 12 h body temperature increase from baseline during rest phase was more pronounced in RW than in AW on work days three and four (*p* < 0.02 both), and lower during the 12 h active phase on work day four (*p* = 0.02). 

In AW, total 24 h food intake was reduced on work days one and two compared to baseline (*p* < 0.05, both days), but not on work days three to four (Figure 2a). During the 8 h work sessions, food intake was only significantly reduced on work day three compared to baseline (*p* < 0.001, Figure 2b). Food intake in the 16 h between work sessions was not significantly different from baseline.

AW body weight was reduced across 24 h (*p* < 0.001, all workdays) and during the 8 h work sessions (*p* < 0.001, all work sessions), but increased more during the 16 h between the work sessions (*p* < 0.001, all workdays) compared to baseline condition (Figure 3). 

Mean 24 h body temperature during the four-day work period in AW was slightly elevated (0.17 °C on average), but not significantly different from baseline (Figure 4a). AW mean 12 h body temperature during the active phase (including the 8 h work session) was unaltered compared to baseline. Mean 12 h body temperature during the rest phase between work sessions was increased by 0.31 °C on average compared to baseline (*p* < 0.001, all days).

### 3.3. The Recovery Period

**Figure 5:**
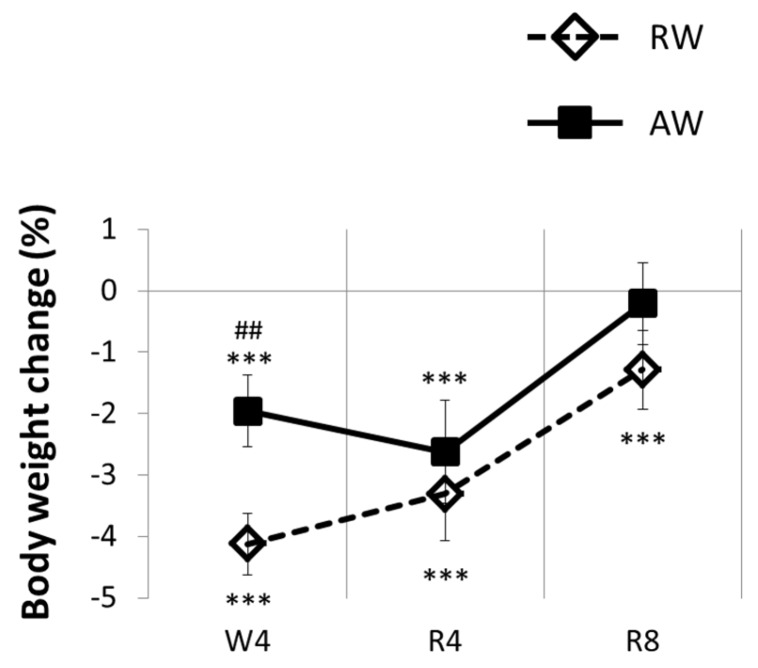
Body weight change during the recovery period for active workers (AW) and rest-workers (RW). Data are shown as percentage change relative to baseline. Error bars indicate SEM. W4 indicates work day. R4–8 indicates recovery days 4 and 8. *** *p* < 0.001, compared to baseline; ## *p* < 0.01, between groups.

Rest-workers increased but did not regain the lost body weight during the 8 days recovery phase (*p* < 0.001; Figure 5). Mean body temperature for 24 h and for 12 h active phase was not significantly different from baseline, while mean body temperature during the 12 h rest phase progressively declined in the course of the recovery period, until it was significantly lower than baseline on recovery days five to eight (*p* < 0.02; Figure 4b). 

Active-workers gradually increased their body weight, and returned to baseline level in the course of the eight-day recovery period (Figure 5). On recovery day four, body weight was still significantly lower than baseline (*p* < 0.001), but not on recovery day eight (*p* = 0.20). Mean values of body temperature in the recovery period did not differ from baseline.

### 3.4. Metabolic Gene Expression in the Liver at the Shift in Feeding Rhythm of Rest-Workers

**Figure 6:**
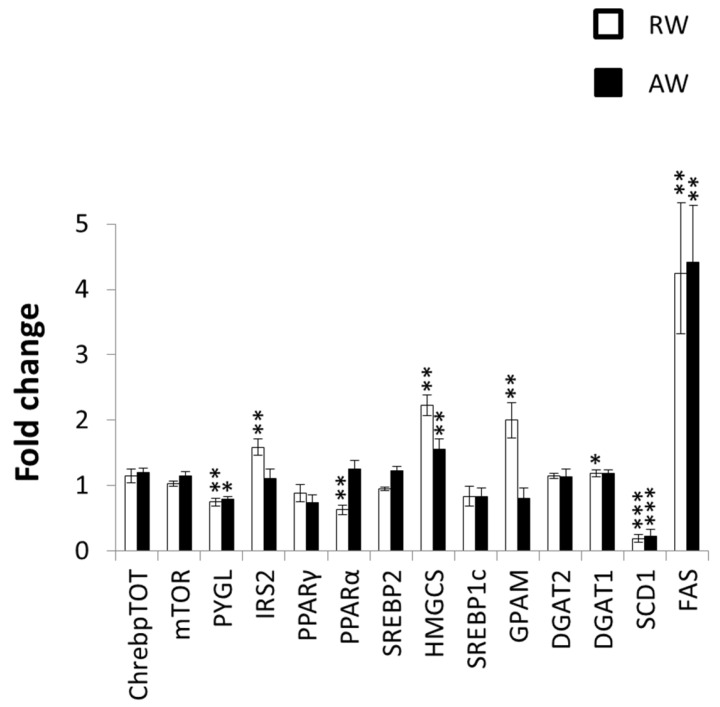
Expression levels of key metabolic genes in the liver from rest-workers (RW) and active workers (AW) following three work sessions, compared to undisturbed rats never exposed to simulated shift work. AW (*n* = 10) were sacrificed at zeitgeber time (ZT)24, and RW (*n* = 10) at ZT12. Undisturbed rats (RW control (*n* = 5) and AW control (*n* = 5) were sacrificed in a time-matched manner (i.e., at the same zeitgeber times as experimental animals). Fold changes for each gene represent the relative difference between shift work rats and controls. A fold change of 1 means no difference compared to controls, while a fold change of 2 means a doubling of transcriptional level, and a fold change of 0.5 a halving of the transcriptional level. * *p* < 0.05; ** *p* < 0.01; *** *p* < 0.001. ChrebpTOT, Carbohydrate-responsive element-binding protein; mTOR, Machanistic target of repemycin; PYGL, Phosphorylase, glycogen, liver; IRS2, Insulin receptor substrate 2; PPAR, Peroxisome proliferator-activated receptor; SREBP, Sterol regulatory element-binding protein; HMGCS, Hydroxymethylglutaryl-CoA synthase; GPAM, Glycerol-3-phosphate acyltransferase 1 mitochondrial; DGAT, Diacylglycerol O-acyltransferase; SCD1, Stearoyl-CoA desaturase 1; FAS, fatty acid synthase.

In our protocol, AW and RW animals were euthanized on opposite zeitgeber time points, which allowed for comparison to time-matched controls only. Transcriptional alterations in several key genes involved in glucose metabolism, insulin sensitivity, fatty acid synthesis, triglyceride synthesis, cholesterol synthesis, and fatty acid oxidation were present in RW compared to undisturbed, time-matched control animals (Figure 6). mRNA levels for the genes encoding Insulin receptor substrate 2 (*Irs2*), Hydroxymethylglutaryl-CoA synthase, cytoplasmic (*Hmgcs1*), and Glycerol-3-phosphate acyltransferase 1, mitochondrial (*Gpam*) were all upregulated in RW compared to time-matched controls. Less-pronounced upregulation of *Hmgcs1* was present in AW, compared to time-matched controls. Transcriptional levels of Fatty acid synthase (*Fas*) were upregulated four-fold both in RW and in AW, while transcription of Stearoyl-CoA desaturase 1 (*Scd1*) was significantly downregulated in the liver from both groups. Transcription of Peroxisome proliferator-activated receptor alpha (*Ppara*, a major regulator of fatty acid oxidation) was downregulated in RW, but not in AW.

## 4. Discussion

The aim of this study was to examine how one period of simulated night-shift work affects metabolism in male rats. Results show that three successive days, each with 8 h of forced activity, led to a shift in the feeding rhythm during the rest-work sessions in rats. Measures of food intake and body weight appear to partially stabilize after three days of rest-work, indicating an adaptation in the regulation of metabolism at this time point.

In this study, both RW and AW exhibited an overall reduction in food intake during the four-day work period. Other studies in animals report inconsistent results regarding food intake during day- and night-shift work. A review of animal models of shift work and effects on metabolism found that effects depended on the species examined (rat or mouse) and the protocol used (manipulate timing of activity, light, food intake, and/or sleep) [21]. In human night workers, a recent meta-analysis reported no change in total calorie intake [26], although there are many other documented changes, such as timing of food intake, number, size, and nutritional content of meals [27]. 

Our data demonstrated that the metabolic gene expression after three days of work was more affected in RW than in AW. Correspondingly, a previous study concluded that following a long period (five weeks) of forced shift work, synchronization of clock genes (controlled by the SCN) and metabolic genes was lost in the liver of RW rats, primarily due to a shift in food intake towards the rest phase [28]. We found that the gene encoding *Irs2* (which is linked to liver insulin sensitivity and lipid metabolism) was upregulated in RW, in correspondence with reduced total food intake [29,30,31]. Upregulation of the gene encoding the central cholesterol-producing enzyme *Hmgcs1* could also be related to reduced food intake, with SREBP2 activation triggered by reduced sterol levels in the liver. However, the genes encoding *Fasn*—the rate-limiting enzyme in de novo fatty acid synthesis—and *Gpam*--which catalyses the first acylation step in the synthesis of glycerophospholipids—were also upregulated in RW. Such upregulation would not be expected to accompany decreased food intake [32]. The increased hepatic expression of these genes in RW may be indicative of paradoxical or compensatory fatty acid and triglyceride synthesis in the liver, via as-of-yet undisclosed molecular mechanisms. In addition, the reduced expression of *Ppara*—indicating decreased fatty acid oxidation—is paradoxical. This uncoupling between food intake, body weight, and lipid biosynthesis/oxidation underlines the metabolically-disruptive potential of rest-work. 

While these data on liver gene expression clearly suggest effects of rest phase work, the results should be considered with some care, for several reasons. First, liver gene expression was measured at only a single time point after three work days in both the AW and RW groups. Since time of day was controlled for by comparing gene expression of working animals to undisturbed time-matched controls, the results do not reflect effects of zeitgeber time only, but more probably reflect the effects of forced activity. Nevertheless, a more detailed study is required to understand the temporal dynamics of these changes in gene expression, not only in the course of the four-day work period, but also during recovery thereafter. Second, in our current protocol, animals were fasted from 2 h before euthanasia, but this may not fully exclude the possibility that our gene expression data were affected by difference in food intake prior to tissue harvesting. Additionally, for this reason, future studies are required to assess in more detail the complex interactions between food intake, metabolic state, and gene expression in the liver. Moreover, in order to examine the significance of transcriptional alterations, relevant protein levels and metabolites such as glucose, insulin, and lipids should be investigated in future studies.

Very few animal studies report or discuss immediate early metabolic effects of night-shift work. Some studies present day-by-day body weight data visually, and appear to indicate a shift in metabolic (body weight) regulation following 2–3 days of simulated night-shift work, but this shift is generally not acknowledged or discussed [33,34,35]. Our data demonstrated that AW maintained a normal rhythm of food intake during the work period, consuming most of the food during their usual active phase, whereas RW gradually shifted timing of food intake towards working hours in their normal rest phase, with a stable high food intake reached and maintained at the third work session. A shift in timing of food intake is consistent with previous findings in the laboratory rat [36]. In parallel, human night-shift workers typically consume the majority of their calories during shifts [37]. Interestingly, the shift in food intake occurred between work days two and three in our study—a time window that parallels the shift in metabolism-related processes during simulated night-shift in humans [20,38]. 

In accordance with the overall reduction in food intake, a net weight loss was observed during the shift work period. Weight loss was more evident in RW than AW, and although body weight increased sharply during the eight-day recovery period, RW did not fully recover body weight to baseline level. Previous studies have reported both weight gain and weight loss during simulated rest-work [34,36]. However, in these studies, rats were exposed to much longer periods of rest-work (4–5 weeks), making it difficult to directly compare our short-term results. Additionally, in the present study, simulated work was achieved with wheels rotating at a faster pace than in previous studies (e.g., [34,36]), which might explain a more negative energy balance. Still, the rats in our study were forced to walk only approximately 1.1 km each work session. Although this physical activity is not considered strenuous, as rats with free access to a running wheel may voluntarily run 6–9 km each day [39], the work sessions may have contributed to weight loss through increased energy expenditure. Such increased energy expenditure might be a direct consequence of the increased physical activity, but it might also partly depend on indirect mechanisms, such as an elevated body temperature. Intriguingly, this presumed increase in energy expenditure was not compensated for by food intake.

While the rats in the RW group consumed more food during the 8 h work sessions than they normally would during the same phase of the day under baseline conditions, they reduced food intake during the 16 h period between sessions (the net result being a reduction in 24-h food intake). Despite this reduction in energy intake between work sessions, body weight increased between sessions, presumably because the lower energy intake was compensated for by parallel reduction in energy expenditure. In agreement with this, RW body temperature was decreased between sessions, perhaps partly as a consequence of increased sleep [23]. This possibly represents a compensatory mechanism aimed at maintaining body weight. A previous study in humans suggests that two to three days of night-shift work is required for such metabolic compensatory mechanisms to take effect [20]. Thus, it may be that the initial days of night-shift work are the most straining on the metabolic system. Measurements of thermogenesis could have shed light on the energy expenditure gap demonstrated by our results, and should be included in future experiments. 

Mean body temperature levels in AW rapidly normalized on the first day of recovery. Surprisingly, while RW initially recovered from the aberrant mean body temperature patterns displayed during the work period, they developed progressive hypothermia in the rest phase later on in the recovery phase. On recovery day eight, rest phase mean body temperature was almost 0.3 °C lower than at baseline. Since recordings were ended at recovery day eight, we do not know how many days hypothermia lasted, or how severe it became. The long-term effect on body temperature may be due to either stress, metabolic dysfunction, or a combination of both [40,41,42,43]. Based on the unexpected failure of all parameters to return to baseline levels, future studies should aim to prolong recovery time after forced work periods. 

The model applied in the present study inherently entails that RW were exposed to light during work sessions, whereas AW were not. Due to the study design, we are precluded from investigating the contribution of light exposure to the metabolic changes. This should be addressed in future studies. 

## 5. Conclusions

Our results indicate that in male rats, 3–4 days of forced work is sufficient to induce disruption to several metabolic parameters, particularly in rats exposed to forced activity during their rest phase. The current findings suggest that the initial days of night-shift work put the most strain on the metabolic system, inducing compensatory effects after 2–3 days, and that neither body weight nor body temperature is fully normalized in eight days of recovery. 

## Figures and Tables

**Table 1:** Sequence names, accession numbers, and primer sequences for genes examined for hepatic transcriptional levels.

Gene	Main Function	Accession Number	Forward Primer	Reverse Primer
Fatty acid synthase (*Fasn*)	Fatty acid synthesis	NM_017332	CCATCATCCCCTTGATGAAGA	GTTGATGTCGATGCCTGTGAG
Stearoyl-CoA 9-desaturase; Stearoyl-CoA desaturase 1 (*Scd1*)	Fatty acid desaturation	NM_139192	TCAATCTCGGGAGAACATCC	CATGCAGTCGATGAAGAACG
Diacylglycerol O-acyltransferase 1 (*Dgat1*)	Triglyceride synthesis	NM_053437	AATGCTGCGGAAAAACTACG	TTGCTGGTAACAGTGCTTGC
Diacylglycerol O-acyltransferase 2 (*Dgat2*)	Triglyceride synthesis	NM_001012345	AATCTGTGGTGCCGCCAG	TCCCTGCAGACACAGCTTTG
Glycerol-3-phosphate acyltransferase 1, mitochondrial (*Gpam*)	Triglyceride synthesis	NM_017274	AATGCTGCGGAAAAACTACG	TTGCTGGTAACAGTGCTTGC
Sterol regulatory element-binding protein 1c (*Srebf1*)	Key regulator of fatty acid/triglyceride synthesis	NM_001271207	GAACCGCAAAGGCTTTGTAAA	ACCCAGATCAGCTCCATGGC
Hydroxymethylglutaryl-CoA synthase, cytoplasmic (*Hmgcs1*)	Sterol synthesis	NM_017268	CAGCTCTTGGGATGGACGA	GGCGTTTCCTGAGGCATATATAG
Sterol regulatory element-binding protein 2 (*Srebf2*)	Key regulator of sterol synthesis	NM_001033694.1	GCCGCAACCAGCTTTCAA	CCTGCTGCACCTGTGGTGTA
Peroxisome proliferator-activated receptor alpha (*Ppara*)	Fatty acid oxidation	NM_013196	AATGCAATCCGTTTTGGAAGA	ACAGGTAAGGATTTCTGCCTTCAG
Peroxisome proliferator-activated receptor gamma (*Pparg*)	Adipocyte differentiation	NM_001145366	CCACAAAAAGAGTAGAAATAAATGTCAGTAC	CAAACCTGATGGCATTGTGAGA
Insulin receptor substrate 2 (*Irs2*)	Mediation of insulin effects	NM_001168633	GAAGCGGCTAAGTCTCATGG	CTGGCTGACTTGAAGGAAGG
Phosphorylase, glycogen, liver (*Pygl*)	Glycogen breakdown	NM_022268	AAAAGCCTGGAACACAATGG	TCGGTCACTGGAGAACTTCC
Mechanistic target of rapamycin (*Mtor*)	Mediation of cellular metabolic stress response	NM_019906	CATGAGATGTGGCATGAAGG	AAACATGCCTTTGACGTTCC
Carbohydrate responsive element binding protein (ChREBP/Mlxipl)	Triglyceride synthesis in response to carbohydrates	AB074517	CTCTCAGGGAATACACGTCTCC	ATCTTGGTCTTTGGGTCTTCAGG
β-actin (*Actb*)	Endogenous control	NM_031144	TACAGCTTCACCACCACAGC	CTTCTCCAGGGAGGAAGAGG
*Rattus norvegicus* ribosomal protein lateral stalk subunit P0 (*Rplp0*)	Endogenous control	NM_022402.2	CATTGAAATCCTGAGCGATGT	AGATGTTCAACATGTTCAGCAG
